# Theoretical Insights into the Oxidative Stress-Relieving Properties of Pinocembrin—An Isolated Flavonoid from Honey and Propolis

**DOI:** 10.1021/acs.jpcb.3c03545

**Published:** 2023-10-10

**Authors:** Maciej Spiegel

**Affiliations:** Department of Pharmacognosy and Herbal Medicines, Wroclaw Medical University, Borowska 211A, 50-556 Wroclaw, Poland

## Abstract

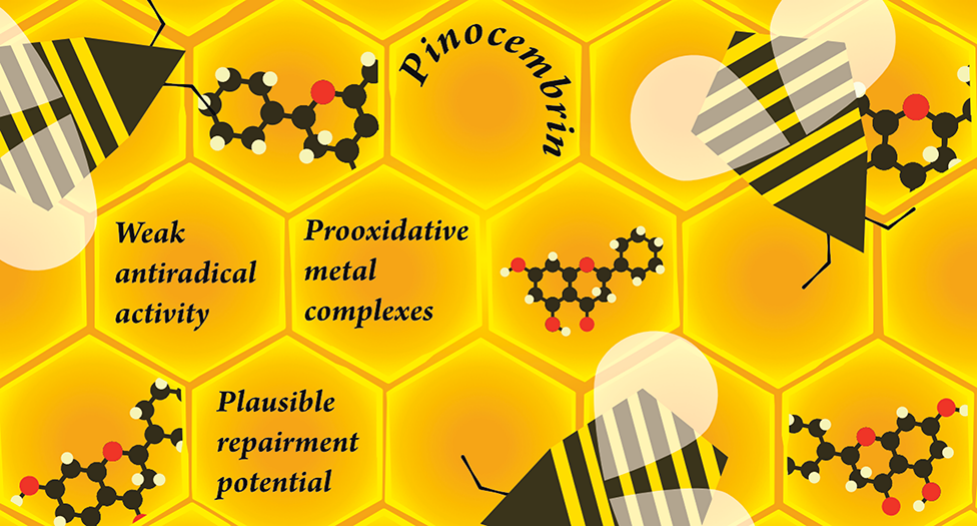

Bee products are a valuable group of substances that have a wide range of applications for humans. They contain a high level of polyphenolic compounds, which have been shown to combat radicals and effectively reduce oxidative stress. In this study, density functional theory was utilized to determine the anti-OOH activity, sequestration of free Cu(II) and Fe(III) ions, the potential pro-oxidative activity of the formed complexes, and the repairing capabilities toward essential biomolecules. The kinetic constants for scavenging of hydroperoxide radical were found to be low, with an order of magnitude not exceeding 10^–3^ M^–1^ s^–1^. Chelating properties showed slightly more satisfactory outcomes, although most complexes exhibited pro-oxidant activity. Pinocembrin, however, proved effective in repairing oxidatively damaged biological compounds and restoring their original functionality. The study found that whilst the system displays limited type I and type II antioxidant activity, it may support the role of physiological reductants already present in the biological matrix.

## Introduction

Although there are 20,000 bee species worldwide , the Western honeybee (*Apis mellifera*) is one of the few bred on a mass scale globally. Bees have been domesticated for millennia to collect their daily produce, including honey, propolis, wax, pollen and royal jelly. Recent studies have revealed the positive impacts of consuming the first two. In clinical trials, it was observed that patients who consumed a daily dose of diluted honey experienced a significant decrease in the levels of prostaglandins E2 and F2a as well as thromboxane B2.^1^ Despite its known sweetness, honey has been found to stimulate Langerhans β-cells and promote insultion secretion, thereby lessen type 1 diabetes.^2^ Additionally, propolis has been demonstrated to influence glycaemic parameters, including HbA1C, FSG, FPG,^3^ and the ApoB/ApoA-I ratio,^4^ thus exhibiting efficacy against type 2 diabetes. These properties have been attributed to the antioxidant properties of its constituents.^5^ Although the composition of propolis and honey varies not only between themselves^6^ but also based on their geographical origin,^7−9^ both are abundant in polyphenolic compounds.

These phytochemicals are an essential class of natural products due to their antiradical and antioxidant potential, resulting in pleiotropic pharmacological effects. Their beneficial activities have been thoroughly investigated, mainly concerning their influence on lipid profile, blood pressure, overall vascular and endothelial function, as well as therapeutic effects on various ailments, such as cancer, osteoporosis, and diabetes.^10^ Polyphenolic antioxidants neutralize free radicals, which are unstable molecules that can damage cells and contribute to the development of diseases. For example, they are involved in the formation of amyloid plaque in the progression of Alzheimer’s disease^11^ or the destruction of dopaminergic neurons in the case of Parkinson’s.^12^ Additionaly, antioxidants help protect DNA from lesions caused by, for instance, the oxidation of guanine to 8-oxoguanine, which can lead to severe mutations and promote cancer development.^13^ Finally, by restraining the expression of pro-inflammatory cytokines such as interleukin-1β (IL-1β), interleukin-6 (IL-6), and tumor necrosis factor-alpha (TNF-α),^14^ they weaken the inflammatory response in atherosclerosis.^15^ Simultaneously, polyphenols can boost the generation of the anti-inflammatory interleukin-10 (IL-10),^16^ which assists in neutralizing the pro-inflammatory response and triggering recovery in affected arteries. The advantageous function of polyphenolic antioxidants appears so cutting-edge that multiple structural alterations and novel delivery approaches have been suggested to enhance their bioavailability^17^ and bioactivity.^18^

At the atomic level, polyphenols are capable of quenching free radicals through a number of mechanisms. The QM-ORSA protocol,^19−21^ which is renowned in antioxidant research, outlines three primary processes by which this is achieved: (1) formal hydrogen atom transfer (*f*-HAT) involves direct donation of a hydrogen atom; (2) Single-electron transfer (SET) entails the transfer of an electron to a radical species (this mechanism is particularly effective in scavenging biologically relevant reactive oxygen species (ROS) such as superoxide radicals, hydroxyl radicals, and singlet oxygen);^22,23^ During (3) radical adduct formation (RAF), an antioxidant forms an adduct with a radical, reducing its reactivity.

Beside these activities, often referred to as type I, there are two other ways in which polyphenols can prevent oxidative damage. One such mechanism is chelation of transition metals (type II activity), typically copper and iron, that partake in the Fenton reaction, leading to the formation of hydroxyl radicals. Such metal binding generally impedes interaction with O_2_^•–^ in redox processes, as confirmed by reaction rate constants.^24^ The third approach to action (type III) centers on preventing the formation of free radicals *in vivo* through blocking enzymes responsible for their generation during catalytic activity. Inducible nitric oxide synthase,^25^ lipoxygenase^26^ and cyclooxygenase^27^ are the ones that might be listed as examples. Furthemore, it should be noted that antioxidants might also be able to repair already damaged biological targets, restoring their physiological role.

**Figure 1:**
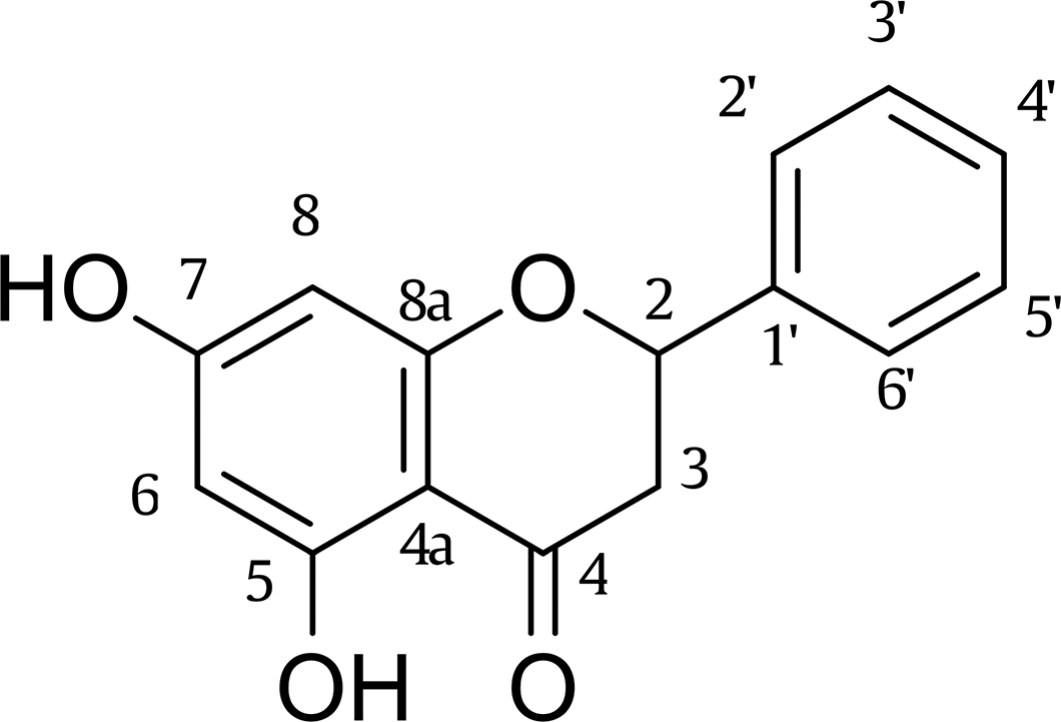
Chemical structure of pinocembrin.

Flavonoids are the most noteworthy polyphenolics. Pinocembrin (Figure 1), pinobanksin, and pinostrobin, which are identified in propolis and honey, are among these substances. These pharmacologically active species demonstrate anti-inflammatory, antibacterial, and neuroprotective properties through their antioxidative and antiradical activity.^28,29^ It is of particular interest in the context of pinocembrin, since it not only belongs to the the subclass of flavanones and thus lacks the crucial delocalization effect between the heterocyclice pyrane and the side ring,^30^ but also has only two hydroxyl groups located in the relatively less active A ring.^31^ Yet, experimental data from DPPH decoloration assays points its antiradical activity.^32^ In fact, its inhibitory properties against xanthine oxidase, cyclooxygenase type-2 and other inflammation-associated enzymes are also heralded.^32,33^

Computational studies were conducted utilizing density functional theory to scrutinize the substance and offer data on its activities from a quantum-mechanical perspective. These activities encompassed primary scavenging activity against ^•^OOH radical and secondary, such as the sequestration of Fe^3+^ and Cu^2+^ ions. It was also examined for its ability to repair critical biomolecules. Besides, the special emphasis was placed on assessing whether the structures lacking hydroxyl groups and of a weakly delocalized electron cloud could still unveil favorable antioxidant activity.

## Computational Methods

The low-energy ground-state conformer of neutral pinocembrin was generated by employing a rigorous conformer search procedure that drawson metadynamic sampling and z-matrix genetic crossing, iMTD-GC, as implemented in the CREST driver program.^34^

Quantum-mechanical investigations were conducted within the framework of density functional theory using the Gaussian16 software package.^35^ The electronic, thermochemical, and kinetic properties of the systems were determined by means of the hybrid meta exchange–correlation functional M05-2X,^36^ as it has been and continues to be considered particularly suitable for the study of radical scavenging activity by polyphenols.^37,38^ The Pople’s 6-311+G(d,p) basis set^39,40^ was chosen as a fairly good compromise between the uptake of computational resources and the accuracy of the outcomes obtained. A universal solvation model based on solute electron density (SMD) which is optimized for the chosen functional,^41^ was picked to assess the examined characteristics in biologically relevant surroundings including water (ε = 78.5) and pentyl ethanoate (ε = 4.7; mimicking membrane lipids, referred to as PE). At every point in the study, the absence of imaginary modes (local minima) or the presence of only one mode that corresponds to the anticipated motion along the reaction path (transition states) was ascertained. Furthemore, intrinsic reaction coordinate calculations were carried out to ensure that the intercepted transition state adequatly links the two corresponding energy minima. All computations were performed at a temperature of 298.15 K, and the unrestricted formalism was applied to radical species.

To account for the presence of differently ionized species in a polar solvent at physiological pH, the fitting parameters method^42^ was used to obtain p*K*_a_ values. Species with non-negligible molar fractions (^M^*f* > 0.1%) were included in the study to ensure reliability of its outcomes. Previous studies have demonstrated that species with low fractions may still play a crucial role in determining activity.

At the outset, the electronic structure was investigated through intrinsic reactivity indices comprising of bond dissociation energy (BDE, eq 1), adiabatic ionization potential (IP, eq 2), proton dissociation energy (PDE, eq 3), proton affinity (PA, eq 4), and electron transfer energy (ETE, eq 5). Their results providing a general outlook in species activity. Although they do not offer precise data on radical scavenging reactions, they give an overview of species' activity in general and are useful for comparative purposes. The energies of solvated electrons and protons were obtained from the paper of Marković et al.^43^
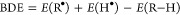
1
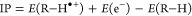
2
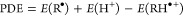
3
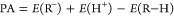
4
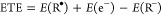
5

To assess the antiradical potential, the reactions of interest, such as formal hydrogen atom transfer (*f*-HAT, eq 6), radical adduct formation (RAF, eq 7), and single electron transfer (SET, eq 8) were examined. As mentioned previously, they depict the primary mechanisms that antioxidants undergo when counteracting free radicals. The relevant Gibbs free energies for kinetic studies were obtained in the following manner:
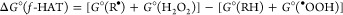
6
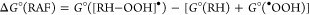
7
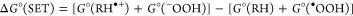
8

While the hydroxyl radical, ^•^OH, is widely recognized as the primary initiator of oxidative damage, it high reactivity means that it quickly reacts, at a diffusion-limited rate, with almost any molecules in its proximity before an antioxidant can intercept it. This renders theoretical studies to yield high reaction rates that are difficult to properly interpet in the context of activity. To overcome this issue, the ^•^OOH radical was chosen because it has a long enough half-life to be intercepted before oxidation of biological targets occurs. Moreover, although the p*K*_a_ value of the ^•^OOH/O_2_^•–^ pair is 4.8, indicating that the deprotonated form is predominant under physiological conditions (0.25% vs 99.75%), the O_2_^•–^ ion carries a negative charge, making its electronic structure disinclined to acquire an additional electron. As a nucleophile and mild reducing agent, it possesses minimal impact on biological targets.^44,45^ Thereby, its protonated form is considered a primary contributor to oxidative damage, despite its significantly lower molar fraction.^46^

To obtain rate constants, the QM-ORSA protocol^19^ based on conventional transition state theory (eq 9) and Marcus theory for electron-related reactions (eq 10)^47^ were applied. For reactions near the diffusion limit, the Collins–Kimball theory (eq 11),^48^ the steady-state Smoluchowski rate coefficient (eq 12),^49^ and the Stoke–Einstein approach to assess mutual diffusion coefficient (eq 13)^50^ were employed. The reaction rate correction stemming from the hydroperoxide molar fraction of equilibrated ^•^OOH ⇄ ^–•^O_2_ in water solvent (0.25%) was also taken into account.
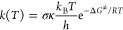
9
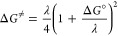
10
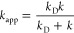
11
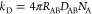
12
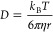
13Details of the mathematical formulations are available elsewhere.^19^

The metal chelating abilities of pinocembrin were assessed using the M05 functional,^51^ which is parametrized for both metals and non-metals, in contrast to M05-2X.^36^ Due to their involvment in the Fenton reaction, Cu^2+^ and Fe^3+^ were the focus of the analysis. For optimal precision, their electrons were described utilizing full electron 6-311+G(d,p) basis functions. Mono- and bidentate complexes were investigated for the C_4_C_5_ scaffold of pinocembrin This motif is the sole site capable of chelating metals (eqs 14 and 15). The ability of the species to bind the ions was determined by complexation constants (eq 16), and the pro-oxidant activity of the resulting complexes was assessed by examining the feasibility and kinetic parameters of their reduction pathways (Fe(III)-to-Fe(II),eq 17); Cu(II)-to-Cu(I),eq 18) induced by biological reductants such as ascorbate or superoxide anion.
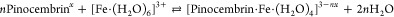
14
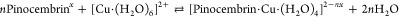
15
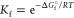
16
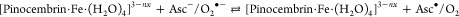
17
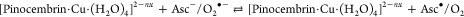
18

The reparative capabilities of the substance on a range of vital biomolecules were assessed using a recently proposed computational procedure.^52^ This involved studies on a simplified linoleic acid model that represents unsaturated fatty acids, six *N*-formyl derivatives of amino acids that are highly vulnerable to oxidative damage, such as Cys, Leu, Tyr, Trp, Met, and His, and DNA fragments of 2′-deoxyguanosine, which are the nucleosides most easily oxidized. Antioxidants are able to use either the SET or *f*-HAT mechanisms, or a combination of both, to restore and regenerate these biomolecules.

## Results and Discussion

### Acid–Base Equilibria

Despite the compound having only two hydroxyl groups, neutral and monoanionic species are anticipated to coexist in water at a pH of 7.4. This is due to two factors: (1) deprotonation of C_7_ often occurs before reaching physiological pH;^22,24,38,53^ (2), C_5_–OH, which can participate in an intramolecular hydrogen bond with an adjacent carbonyl oxygen, indirectly contributes to density delocalization, thereby stabilizing the system and reducing the tendency for dissociation.^31^

**Figure 2:**
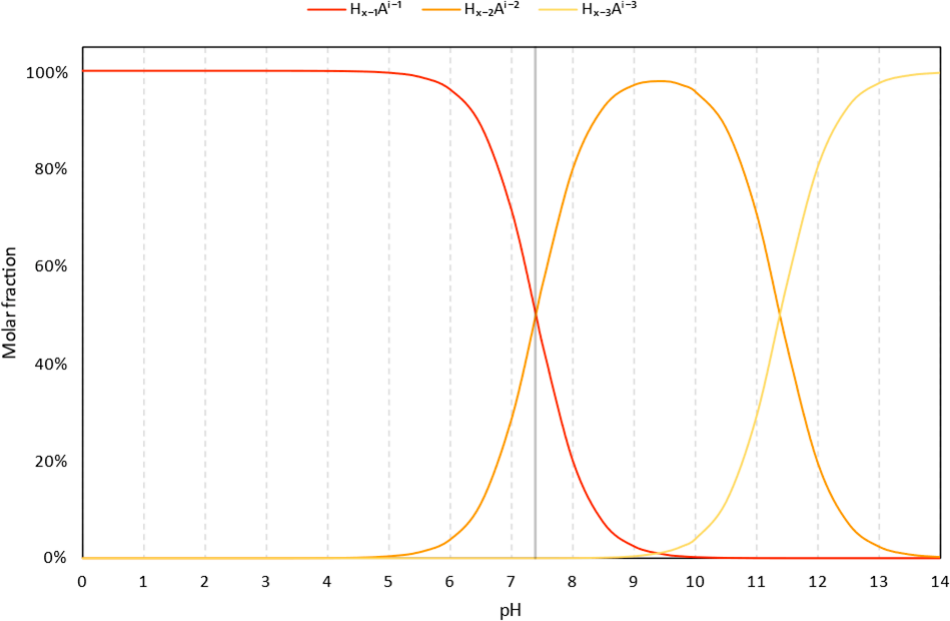
Molar fractions of pinocembrin species plotted as a function of pH.

The initial dissociation indeed takes place at C_7_, which has a p*K*_a1_ value of 7.40. The deprotonation of the hydroxyl group at C_5_ occurs at significantly higher pH (p*K*_a2_ = 11.38). The outcomes are illustrated in Figure 2, which depicts the molar fraction of each species as pH varies. The monoanionic form is prevalent at physiological pH (); nevertheless, neutral species with  are also expected to contribute substantially to the compound's activity. Dianion is not present under the given conditions and can consequently be excluded from the discussion.

### Intrinsic Reactivity Indices

To preliminarily assess the antiradical potential of the species studied, their intrinsic reactivity indices were established and compiled in Table 1.

**Table 1:** Values of Intrinsic Reactivity Indices Calculated for Pinocembrin Species in Pentyl Ethanoate and Water at pH = 7.4 and 298.15 K^a^

Solvent	Species	Site	BDE	IP	PDE	PA	ETE
pentyl ethanoate	H_2_Pin	C_5_	107.5	139.9	23.9	75.7	88.1
		C_7_	101.9		18.2	61.8	96.3
water	H_2_Pin	C_5_	104.1	121.1	18.6	42.7	96.9
		C_7_	104.7		19.2	37.1	103.1
	HPin^–^	C_5_	101.2	101.2	33.6	49.0	87.7

a BDE – bond dissociation energy. IP – ionization potential. PDE – proton dissociation energy. PA – proton affinity. ETE – electron transfer energy. All values are given in kcal mol^–1^.

The outcomes of the bond dissociation processes reveal that although the homolytic fission of the O–H bond of C_7_–OH in pentyl ethanoate is slightly more favorable than that of C_5_ by approximately 6 kcal mol^–1^), both positions in water can be viewed as equal with an energy discrepancy of about 0.6 kcal mol^–1^. Furhtemore, when moving from pentyl ethanoate to water, a minor decrease in BDE for C_5_ and a small rise for C_7_ is noticeable. Interestingly, the pattern is now C_7_ > C_5_, which is a reversal of the previous trend. Similarly, deprotonation leads to a slight increase in the process's feasability. Two papers have examined this property in water, but with slightly different levels of theory, M06-2X/6-311+G**/6-31G* (SMD)^54^ and BMK/6-311+G** (C-PCM).^55^ The values estimated for the set (C5, C7) were (94.4, 93.9) and (89.9, 90.1), all in kcal mol^–1^. The results outlined in this Article indicate an increase in values, with a corresponding pattern evident for M06-2X. Conversely, the BMK functional displays the opposite trend. Albeit all of these are GH meta-GGA DFT functionals,^36,56,57^ they differ in terms of formulation and the fraction of HF exchange included. Specifically, M05-2X and M06-2X are based on 19 and 29 parameters, respectively, with 56% and 54% of HF exchange. In contrast, BMK comprises only 42% of HF exchange and is designed for kinetic research, according to the authors.

As expected, upon the transition from pentyl ethanoate (139.9 kcal mol^–1^) to water (121.1 kcal mol^–1^) and after deprotonation (101.2 kcal mol^–1^), the ionization potential value drops, possible due to the solvation of cation radical formed. The dissimilarity between pentyl ethanoate and monoanionic species is nearly 40.0 kcal mol^–1^. In the studies mentioned earlier, IP values were discovered to be 140.7 kcal mol^–154^ and 114.6 kcal mol^–1^.^55^ At this stage, it seems that BMK produces markedly lower values than Minnesota functionals. However, it should not come as a suprise that there are disparities in in determining IP and EA values because they are highly reliant on the functional and basis set employed.^58^

Proton dissociation energy data demonstrate that the easiest proton dissociation trend is identical to the direct O–H bond cleavage in pentyl ethanoate and water. C_7_ (18.2 kcal mol^–1^) undergoes the process most readily in pentyl ethanoate, whereas C_5_ (18.6 kcal mol^–1^) does so in water. As anticipated, although the generation of the cation radical necessitates more energy in the aprotic solvent, the following release of the proton from the ionized structure is equally affordable in both environments. This suggests that the substance is persistent in maintaining the coupling of all all spins. It is intriguing that the PDE indices obtained here are closer to those reported for the BMK functional (18.3 kcal mol^–1^ for C_5_ and 19.3 kcal mol^–1^ for C_7_) than those obtained for M05-2X (12.6 kcal mol^–1^ for C_5_). In any case, the process for the ionic structure is more energetically demanding; the proton dissociation energy is observably higher (33.6 kcal mol^–1^), implying that the hydrogen in C5 is bound to the hydroxyl oxygen much strongly than in any other case.

Even though the proton affinity mechanism is not expected to be operative for pentyl ethanoate, PA values were also determined for it in order to obtain a complete and systematic insight into the intrinsic reactivity indices. The high energy requirements, higher for C_5_ (75.7 kcal mol^–1^) than for C_7_ (61.8 kcal mol^–1^), are not suprising and arise from the the insufficient solvation capacity of the milieu. The estimated values for water closely match those reported for the BMK functional (42.3 kcal mol^–1^ for C_5_ and 37.2 kcal mol^–1^ for C_7_). Similar to the case of PDE, higher values are observed for monoanionic species than for neutral ones, which are linked to the proton affinity of the C_5_ group.

A striking behavior is observed in relation to the electron transfer energy results, where the lowest values were found for the species in pentyl ethanoate. This signifies that in the event of proton detachment in this environment, the resulting anion is less stable than the one formed in an aqueos solvent and is more prone to conversion into a radical. Lastly, the ETE values in water follow the expected pattern of successive decreases.

The BDE values assessed indicate that *f*-HAT is likely the most viable mechanism in most species, as they are noticeably lower than IP which determines the propensity for SET. The sole exception is monoanion, for which both are equal and therefore may contribute equally to radical scavenging potentials. Nonetheless, intrinsic reactivity indices mostly serve comparative purposes for substances tested at the same level of theory. Antiradical activity should not be considered a given, especially in electron transfer processes. Pinocembrin may be a weaker radical scavenger than apigenin,^38^ scutellarin and scutellarein,^22,53^ or malvidin and its glycosides,^68^ for which both were notably lower.

### Anti-^•^OOH Scavenging Potential

The ability of pinocembrin to intercept the ^•^OOH radical in both tested media was evaluated. Within the framework of computed Gibbs free energies (Δ*G*°) and activation energies (Δ*G*^‡^), the feasability of the three possible processes (*f*-HAT, RAF and SET) was determined. The outcomes are presented in Table 2.

**Table 2:** Thermochemical Data for the Reaction of Pinocembrin with ^•^OOH in Pentyl Ethanoate (Labeled with Superscript ^PE^) and in Water at pH = 7.4 and 298.15 K^a^

		H_2_Pin^PE^		H_2_Pin		HPin^–^	
Mechanism	Site	Δ*G*°	Δ*G*^‡^	Δ*G*°	Δ*G*^‡^	Δ*G*°	Δ*G*^‡^
*f*-HAT	C_5_	17.1		10.7		7.8	43.3
	C_7_	11.6		11.2			
RAF	C_4_	34.6		33.9		14.6	
	C_5_	25.6		22.4		22.2	
	C_6_	24.1		23.8		19.1	
	C_7_	26.0		24.8		32.2	
	C_8_	23.9		22.9		19.2	
	C_1′_	24.0		22.3		22.6	
	C_2′_	22.5		20.9		21.8	
	C_3′_	24.0		22.2		22.4	
	C_4′_	23.3		22.3		22.2	
	C_5′_	23.3		22.4		22.4	
	C_6′_	22.8		21.8		22.2	
SET				40.8	49.3	22.2	23.7

a Δ*G*° – Gibbs free energies. Δ*G*^‡^ – activation energies. All values are given in kcal mol^–1^.

For the initial two mechanisms, the requirement of favorability was that Δ*G*° < 10.0 kcal mol^–1^. This is due to the fact that despite the reversibility of endergonic pathways, they can still be extremly advantageous if subsequent reactions of the resulting products with surrounding substances are highly exergonic, resulting in a driving force and low activation barriers. Such behavior is expected in complex biological systems.^59−61^ In contrast, electron-related processes conform to Marcus' theory^47,62,63^ and thus all merit investigation.

The findings suggest that the hydroxyl group bonded to the C_7_ in pentyl ethanoate has the lowest Gibbs free energy (11.6 kcal mol ^–1^). However, when in water, the trend shifts toward hydrogen abstraction from C_5_ (10.7 kcal mol ^–1^). The impact of deprotonation is also observed to further reduce the given energy by 2.9 kcal mol^–1^, thus C_5_ becoming the sole thermodynamically favorable pathway. The activation energy is particularly high, however, so this process is not expected to be associated with significant antiradical activity. This highlights the impact of environmental conditions on reactivity,^64^ likely through electrostatic interactions between the solvent and the hydroxyl groups of the species.

Similarly, the Δ*G*° values of all RAF pathways are significantly higher than the mentioned threshold, and capability to seize ^•^OOH is questionable in any scenario.

Finally, the values for Δ*G*° and Δ*G*^‡^ describing SET mechanism are especially those associated with neutral species (40.8 and 49.3 kcal mol^–1^, respectively), but could contribute more to the antiradical potential than the formal hydrogen atom transfer, depending on the reorganization energy. The credible activation energy value obtains substantially higher need for monoanion (23.7 kcal mol^–1^), which is lower than that of *f*-HAT from C_5_, indicates that the former process mainly contributes to the overall antioxidant activity.

**Figure 3:**
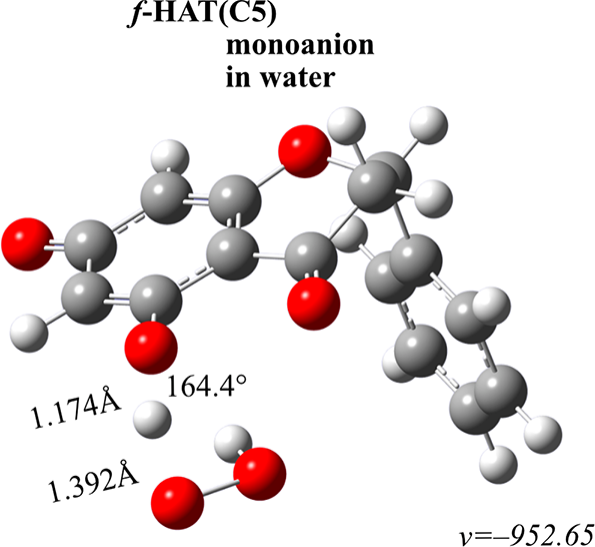
Transition state structure of the viable f-HAT mechanism. Distances are in Å, and angles are in degrees.

The discussion of workable mechanisms is reflected in the rate constants and branching ratios reported in Table 3. Figure 3 depicts the transition state structure of the only feasible *f*-HAT process. While *k*_overall_ proposed in QM-ORSA protocol assumes that the hydroperoxide radical is omnipresent in the solution under study, and hence does not control the reaction kinetics, this is barely true under physiological conditions, leading to the improper conclusions. Therefore, in line with prior research,^38^ a revised kinetic constant was introduced to consider the molar fraction of the radical and enhance the precision of the theoretical data description.^22,53^

**Table 3:** Rate Constants and Branching Ratios (Γ, in %)^68^ for the Viable Reactions between ^•^OOH and Pinocembrin in Water at pH 7.4 and 298.15 K^a^

		H_2_Pin		HPin^–^	
Mechanism	Site	*k*	Γ	*k*	Γ
*f*-HAT	C_5_			3.23 × 10^–19^	0.0
SET		4.19 × 10^–24^	100.0	2.52 × 10^–5^	100.0
*k*_total_		4.19 × 10^–24^		2.52 × 10^–5^	
*k*_overall_		2.08 × 10^–24^		1.27 × 10^–5^	
*k*_corrected_		5.21 × 10^–27^		3.16 × 10^–8^	

a *k* – thermal rate constant. *k*_total_ – sum of all rate constants for a given species. *k*_overall_ – *k*_total_ multiplied by the molar fraction of the species at pH = 7.4. *k*_corrected_ – *k*_overall_ multiplied by the molar fraction of ^•^OOH under the given conditions. Kinetic constant values are given in M^–1^ s^–1^.

The findings show that pinocembrin displays relatively low antiradical activity. The high activation energy value led to the *f*-HAT pathway being disregarded. In the same way, although SET is the primary mechanism of action, the rate constants are not satisfactory, as they do not exceeed the value of 2.52 × 10^–5^ M^–1^ s^–1^ obtained for HPin^–^ species. With regard to neutral form, the value is even lower at 4.19 × 10^–24^ M^–1^ s^–1^. Given the concentration of the hydroperoxide radical, these processes decelerate by approximately three orders of magnitude.

The presented data demonstrates that pinocembrin exhibits significantly lower reactivity than other antioxidants, for example apigenin,^38^ where deprotonation led to an increased rate constants. The rates are even more implausible when compared to commonly used reference substances, such as Trolox^59^ or vitamin C.^65^ Ultimately, the reaction rate constants highlight the crucial aspects of the discussion about intrinsic reactivity indices and confirm their function as a basic yet qualitatively correct technique to compare various antioxidants. However, it is impossible to employ only them for evaluating the propensity of the mechanisms.

It is important to note that comparing the results with the kinetic data of *chrysin* (a flavone, having a C_2_=C_3_ double bond), *pinobanksin* (a flavanonol, having a C_3_–OH group), and *galangin* (a flavonol having both structural motifs) would provide valuable insight into structure–activity relationships. This type of studies is of exceptional merit in the study of antioxidant systems and the modeling of new ones.

### Chelating Properties

Most polyphenols have also the ability to prevent the formation of radical in addition to their type I antioxidant activity. This is accomplished by chelating ions like Fe(III) and Cu(II) that play a part in initiating chain reactions and generating a bunch of oxygen-centered radicals. Despite having only one site capable of complexation, pinocembrin is able to form mono- and bidentate complexes thanks to the coordinating scaffold made up of the C_4_ carbonyl and C_5_ hydroxyl groups, successfully preventing redox reactions leading the metals reduction.

**Figure 4:**
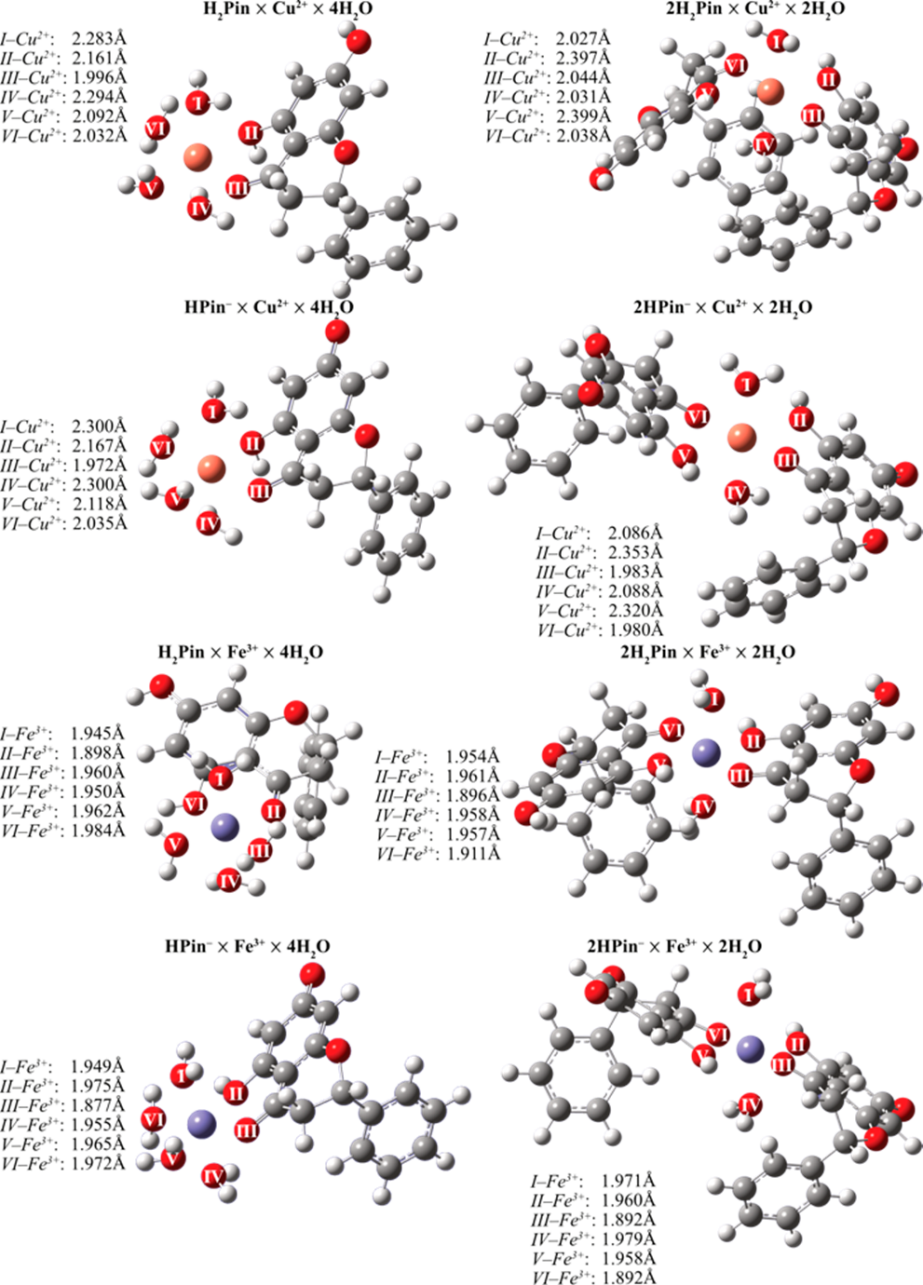
Structures of mono- and bidentate metal complexes. Distances are in Å, and angles are in degrees.

Figure 4 shows that in monodentate systems, the copper ion is chelated at distances of 1.996 Å (neutral) and 1.972 Å (anion) from the carbonyl moiety. In bidentate systems, these distances increase to (2.044 Å, 2.038 Å) (neutral) and (1.983 Å, 1.980 Å) (anion), respectively. As for the C_5_ hydroxyl’s oxygen, the values are only slightly larger: 2.161 Å (neutral) and 2.167 (anion) for monodentate systems, and 2.397 Å, 2.399 Å (neutral) and 2.353 Å, 2.320 Å (anion) for bidentate ones. The bond lengths between copper and donor oxygens display a remarkable degree of elongations. This elongation is suggestive of the semicoordinating nature of the metal-ligand interactions. Furthemore, the tetragonal deformation in these complexes caused by the Jahn−Teller effect is evident by the allocation of axial water molecules farther from the metal center.

In contrast, Fe(III) complexes adhere to an octahedral geometry, with fixed distances of 1.898 Å from carbonyl and 1.984 Å from hydroxyl for neutral species, and 1.877 Å from carbonyl and 1.975 Å from hydroxyl in the case of monoanion for monodentate systems. In non-dissociated structures involving two pinocembrin molecules, the measurements are 1.961 and 1.957 Å from hydroxyl and 1.896 and 1.911 Å from carbonyl. The measurements of the dissociated complexes are 1.960 and 1.958 Å from hydroxyl and 1.892 and 1.892 Å from carbonyl, respectively.

The data in Table 4 underline that Fe(III) complexes are generally better stabilized than Cu(II). Additionaly, the deprotonated chelator has a much higher exhibits a much higher capacity, with *K*_i_ values varying by about four orders of magnitude between both mono- and bidentate Cu(II) systems, and up to twelve when comparing bidentate iron complexes to those based on undissociated species. The enhanced stability following the creation of ionic species can be explained by the equalization of the metal center’s positive charge through the flow of electron density from HPin^–^. It is possible that chelation would be even more robust if the C_5_ group were to dissociate. This occurrence may take place only when the pH reaches about 9. Also, such pH alters also the structure of the metal aqua complexes being examined, as they form hydroxides that are chelated in alternative ways. In any event, when molar fractions are near equivalent, the the apparent inhibition constant seems largely caused by the existence of monoanionic species, while the tole of the undissociated species is insignificant.

**Table 4:** Thermochemical and Kinetic Data of Cu(II) and Fe(III) Complexation Processes by Pinocembrin in Ratios 1:1 and 1:2 at pH = 7.4 and 298.15 K^a^

Species	Δ*G*_f_	*K*_f_	*K*_i_^II^	*K*_i_^app^
Monodentate				
Cu(II)				
H_2_Pin	–0.9	4.71 × 10°	2.35 × 10°	3.46 × 10^4^
HPin^–^	–6.6	6.89 × 10^4^	3.46 × 10^4^	
Fe(III)				
H_2_Pin	0.0	1.00 × 10°	4.98 × 10^–1^	1.43 × 10^6^
HPin^–^	–8.8	2.84 × 10^6^	1.43 × 10^6^	
Bidentate				
Cu(II)				
H_2_Pin	–5.0	4.82 × 10^3^	1.20 × 10^3^	5.83 × 10^8^
HPin^–^	–12.8	2.32 × 10^9^	5.83 × 10^8^	
Fe(III)				
H_2_Pin	–1.0	5.43 × 10°	1.35 × 10°	1.11 × 10^12^
HPin^–^	–17.2	4.41 × 10^12^	1.11 × 10^12^	

a *ΔG*_f_ – Gibbs free energy of complexation. *K*_f_ – formation constant. *K*_i_^II^ – *K*_f_ multiplied by the molar fraction of the species at pH = 7.4. *K*_i_^app^ – sum of *K*_i_^II^. Thermochemical values are given in kcal mol^–1^, and complexation constants values are given in M^–1^ s^–1^.

Despite the fact that coordinated metals in these complexes tend to be less amenable to reduction, the process remains feasible. It may result in the formation of radicals , both directly *via* the production of Asc^•^ and indirectly through the creation of a reduced metal that is capable of undergoing the Fenton process. In order to evaluate this pro-oxidative behavior, the thermochemistry and kinetics of the relevant reactions were examined (Table 5).

**Table 5:** Thermochemical and Kinetic Data for the Mono- and Bidentate Cu(II)-to-Cu(I) Complex Reductions by the Superoxide Anionradical (O_2_^•–^) and Ascorbate (Asc^–^) at pH = 7.4 and 298.15 K^a^

Species	λ	Δ*G*_r_°	Δ*G*^‡^_r_	*k*_ET_	*k*_app_		Species	λ	Δ*G*_r_°	Δ*G*^‡^_r_	*k*_ET_	*k*_app_
[_*i*_L^*j*^ × M(H_2_O)_*n*−2*i*_^*m*^] + O_2_^•–^ → [_*i*_L^*j*^ × M(H_2_O)_*n*−2*i*_^*m*–1^] + O_2_												
Cu(H_2_O)_6_^2+^	26.2	–35.6	0.5	2.66 × 10^12^	3.85 × 10^9^		Fe(H_2_O)_6_^3+^	17.3	–33.2	3.7	1.30 × 10^10^	2.99 × 10^9^
[H_2_Pin × Cu(H_2_O)_4_]^2+^	23.5	–32.1	0.8	1.68 × 10^12^	4.15 × 10^9^		[H_2_Pin × Fe(H_2_O)_4_]^3+^	15.7	–33.6	5.1	1.22 × 10^9^	9.44 × 10^8^
[HPin × Cu(H_2_O)_4_]^+^	25.2	–27.7	0.1	5.59 × 10^12^	4.06 × 10^9^		[HPin × Fe(H_2_O)_4_]^2+^	15.6	–28.9	2.8	5.15 × 10^10^	3.83 × 10^9^
[2H_2_Pin × Cu(H_2_O)_2_]^2+^	23.6	–23.6	0.0	6.21 × 10^12^	4.40 × 10^9^		[2H_2_Pin × Fe(H_2_O)_2_]^3+^	15.7	–32.2	4.4	4.02 × 10^9^	2.07 × 10^9^
[2HPin × Cu(H_2_O)_2_]^+^	22.5	–22.4	0.0	6.21 × 10^12^	4.29 × 10^9^		[2HPin × Fe(H_2_O)_2_] ^2+^	17.0	–23.2	0.6	2.39 × 10^12^	4.28 × 10^9^
[_*i*_L^*j*^ × M(H_2_O)_*n*−2*i*_^*m*^] + Asc^–^ → [_*i*_L^*j*^ × M(H_2_O)_*n*−2*i*_^*m*–1^] + Asc^•^												
Cu(H_2_O)_6_^2+^	21.8	–16.3	0.4	3.40 × 10^12^	4.67 × 10^9^		Fe(H_2_O)_6_^3+^	13.0	–16.1	0.2	4.57 × 10^12^	4.70 × 10^9^
[H_2_Pin × Cu(H_2_O)_4_]^2+^	19.2	–14.9	0.2	4.14 × 10^12^	3.77 × 10^9^		[H_2_Pin × Fe(H_2_O)_4_]^3+^	11.4	–16.4	0.6	2.46 × 10^12^	3.75 × 10^9^
[HPin × Cu(H_2_O)_4_]^+^	20.8	–10.5	1.3	7.18 × 10^11^	3.72 × 10^9^		[HPin × Fe(H_2_O)_4_]^2+^	11.3	–11.7	0.0	6.16 × 10^12^	3.76 × 10^9^
[2H_2_Pin × Cu(H_2_O)_2_]^2+^	19.3	–6.4	2.1	1.68 × 10^11^	3.78 × 10^9^		[2H_2_Pin × Fe(H_2_O)_2_]^3+^	11.4	–15.1	0.3	3.75 × 10^12^	3.80 × 10^9^
[2HPin × Cu(H_2_O)_2_]^+^	18.2	–5.3	2.3	1.30 × 10^11^	3.71 × 10^9^		[2HPin × Fe(H_2_O)_2_] ^2+^	12.7	–6.0	0.9	1.44 × 10^12^	3.80 × 10^9^

a λ – reorganization energy. Δ*G*_r_°– Gibbs free energies. Δ*G*_r_^‡^– activation energies. *k*_ET_ – thermal rate constant. *k*_app_ – *k*_ET_ corrected by diffusion rate constant. Thermochemical values are given in kcal mol^–1^, and complexation constants values are given in M^–1^ s^–1^.

The methodology applied was verified by reference to the experimental reaction rate between copper and superoxide radical, measured by Butler et al.^66^ as (8.1 ± 0.5) × 10^9^ M^–1^ s^–1^ at pH = 7.0 and by Brigelius et al.^67^ as (2.7 ± 0.2) × 10^9^ M^–1^ s^–1^ at pH = 7.8. The established rate of 3.85 × 10^9^ M^–1^ s^–1^ at pH 7.4 lies in between the values reported by the two studies, implying that the results are reliable.

The reduction carried out by the superoxide anion radical seems to be most efficent for copper species with tthermal kinetic constants of magnitude of 12 in all cases. The process is even faster for anionic species or bidentate complexes. Similar activity is observed in the case of iron. Thus, the majority of the formed complexes exhibit slightly greater pro-oxidant potential, as indicated by their apparent rate constants being higher than those of the corresponding aqua complexes, except for [H_2_Pin × Fe(H_2_O)_4_]^3+^ and [2H_2_Pin × Fe(H_2_O)_2_]^3+^.

With respect to ascorbate ions, a comparable pattern is observed regarding the impact of deprotonation and the formation of bidentate systems on the apparent rate constant. Nonetheless, the reduction potential of Asc^–^ toward either of them drops, as seen from kinetic constants lower than those established for the reference compounds. Therefore, a marginal but detectable protective effect can be recognized.

### Repairment Mechanisms

**Figure 5:**
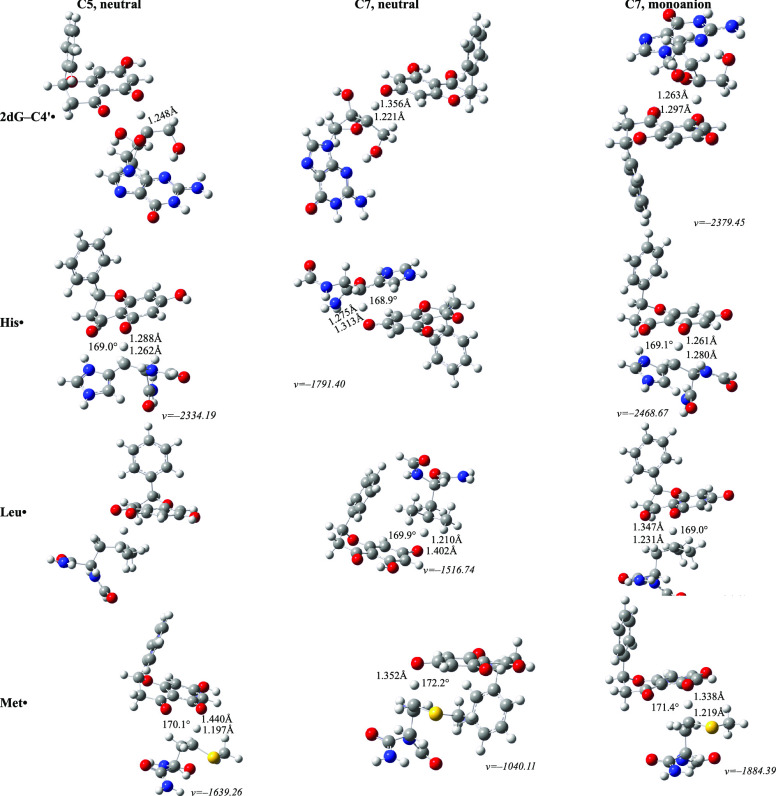
Transition state structures of viable repairment mechanisms. Distances are in Å, and angles are in degrees.

The protective activity of pinocembrin was investigated with regards to its role in repairing damaged biomolecules. A set of pathways were selected and their associated thermochemical and kinetic data are given in Table 6, complemented by the corresponding transition state structures (Figure 5).

**Table 6:** Thermochemical and Kinetic Data of the Repairment Reactions between Pinocembrin and Chosen Biological Targets at pH = 7.4 and 298.15 K^a^

		Δ*G*°	Δ*G*^‡^	*k*	*k*_app_	*k*_total_	*k*_overall_
LM^•^							
H_2_Pin	C_5_	29.5					
	C_7_	23.9					
2dG-C_4′_^•^							
H_2_Pin	C_5_	1.7	30.5	2.87 × 10^–10^	6.25 × 10^–7^	1.05 × 10^–5^	5.46 × 10^–6^
	C_7_	2.2	27.5	4.54 × 10^–8^	1.03 × 10^–5^		
HPin^–^	C_5_	–1.2	31.5	5.39 × 10^–11^	1.07 × 10^–8^		5.36 × 10^–9^
2dG-C_8_^–^OH							
H_2_Pin	C_5_	22.8					
	C_7_	23.3					
HPin^–^	C_5_	19.9					
Cys^•^							
H_2_Pin	C_5_	13.6					
	C_7_	14.0					
HPin^–^	C_5_	10.7					
His^•^							
H_2_Pin	C_5_	9.1	24.6	5.38 × 10^–6^	7.46 × 10^–5^	5.05 × 10^–1^	2.52 × 10^–1^
	C_7_	9.5	17.9	5.05 × 10^–1^	5.05 × 10^–1^		
HPin^–^	C_5_	6.2	23.1	7.69 × 10^–5^	2.75 × 10^–3^		1.38 × 10^–3^
Leu^•^							
H_2_Pin	C_5_	4.6	31.3	6.69 × 10^–11^	3.94 × 10^–9^	3.66 × 10^–8^	1.82 × 10^–8^
	C_7_	5.1	29.3	2.03 × 10^–9^	3.27 × 10^–8^		
HPin^–^	C_5_	1.7	31.5	5.34 × 10^–11^	7.73 × 10^–9^		3.88 × 10^–9^
Met^•^							
H_2_Pin	C_5_	5.7	16.4	5.76 × 10°	5.76 × 10°	3.53 × 10^5^	1.76 × 10^5^
	C_7_	6.1	9.9	3.53 × 10^5^	3.53 × 10^5^		
HPin^–^	C_5_	2.8	17.6	7.26 × 10^–1^	5.99 × 10°		3.01 × 10°
Tyr^•^							
H_2_Pin	C_5_	14.2					
	C_7_	14.7					
HPin^–^	C_5_	11.3					

	λ	Δ*G*_r_	Δ*G*^‡^_r_	*k*_app_	*k*_overall_
2dG^•+^					
H_2_Pin	22.2	10.7	12.2	6.91 × 10^3^	3.44 × 10^3^
HPin^–^	18.6	–7.9	1.5	3.69 × 10^9^	1.85 × 10^9^
Trp^•+^					
H_2_Pin	11.7	18.3	19.2	5.35 × 10^–2^	2.67 × 10^–2^
HPin^–^	8.1	–0.3	1.9	3.68 × 10^9^	1.85 × 10^9^
Tyr^•+^					
H_2_Pin	12.1	6.7	7.3	2.64 × 10^7^	1.31 × 10^7^
HPin^–^	8.4	–11.9	0.3	3.71 × 10^9^	1.86 × 10^9^

a Δ*G*° – Gibbs free energies. Δ*G*^‡^ – activation energies. *k*_app_ – thermal rate constant corrected by diffusion rate constant and Eckart tunneling. λ – reorganization energy. Thermochemical values are given in kcal mol^–1^, and kinetic constants values are given in M^–1^ s^–1^.

Based on thermochemical data, it appears that the pinocembrin species are capable of restoring part of the chosen biomolecules, with the exception of tyrosine and cysteine radicals as well as lesions resulting from the adducts formed between 2dG and a simplified model of linoleic acid with the hydroxyl radical. The Gibbs free energies determined for these exceptions were found to be 11.3, 10.7, 19.9, and 23.9 kcal mol^–1^, respectively, all exceeding the threshold.

Pinocembrin effectively restores the cation radical of tyrosine through electron transfer, regardless of its form, with a react constant of ×10^7–9^ M^–1^ s^–1^. This process is among the most feasible ones. A weaker but still plausible activity can be also be observed with regards to 2dG^•+^ and Trp^•+^. Howevere, it is notably different between these two; while the neutral (*k*_overall_ = 3.44 × 10^3^ M^–1^ s^–1^) and dissociated (*k*_overall_ = 1.85 × 10^9^ M^–1^ s^–1^) forms mitigate the lesions of 2'-deoxyguanosine, only the anionic species is “*kinetically active*” in the case of tryptophan. For the neutral forms, the reaction rate drops drastically to 2.67 × 10^–2^ M^–1^ s^–1^ compared to the ion's 1.85 × 10^9^ M^–1^ s^–1^. This observation is in line with the understanding that anions have a higher affinity for donating electron density than their neutral counterparts.

On the flip side, the process of mending through hydrogen atom transfer appears to be of minor importance. Only methionine can be effectively restored to its biological form in the presence of pinocembrin, and solely when the undissociated species is considered, for which the overall reaction rate was estimated at 1.76 × 10^5^ M^–1^ s^–1^. The anion is noticeably less active, by about a factor of five, rendering it irrelevant. Even smaller values were discovered for reactions with 2dG-C_4′_^•^, His^•^, and Leu^•^.

## Conclusions

Summarizing the collected data, it can be concluded that:

- At a physiological pH, pinocembrin exists in two forms with almost identical mole fractions—neutral and monoanionic. The doubly dissociated species does not exist in these conditions.
- Intrinsic reactivity indices suggested that hydrogen-related processes may make a greater contribution to the overall antiradical activity compared to electron-related ones. However, subsequent findings contradict this notion, thus questioning the validity of these indices as a reliable method for determining antioxidant activity.
- Thermochemical and kinetic studies have shown that pinocembrin lacks any anti-OOH potential in a lipid environment. Moreover, its effectiveness in water is solely reliant upon an electron transfer mechanism, largely due to deprotonation. The antiradical activity of the flavonoid is weaker than that of Trolox, apigenin, or vitamin C.
- Pinocembrin demonstrates moderate chelating properties with selected transition metals in its neutral form, although deprotonation enhances the process. Most of the complexes formed exhibit pro-oxidant potential toward O_2_^•–^. Contrary, the reactions with Asc^*–*^ occur at a slower rate than those with the adequate aqua complexes.
- Even though, the species studied show limited type I and type II activity, they demonstrate a reasonable antioxidative potential, being able to repair several of the oxidatively modified biologically relevant compounds considered susceptible to radical attack.

## Data Availability

All underlying data available in the article itself and its Supporting Information. Additional data can be obtained upon reasonable request.

## Supporting Information Available

Cartesian coordinates of all the studied structures (ZIP)
